# Quantification of Membrane Protein Conformational Free Energy from Mutations and a Single Atom

**DOI:** 10.1021/jacs.5c04065

**Published:** 2025-09-15

**Authors:** Belen Ramirez-Cordero, Nathaniel J. Traaseth

**Affiliations:** Department of Chemistry, 5894 New York University, New York, New York 10003, United States

## Abstract

Secondary active transporters are membrane proteins involved in moving substrates across the cellular membrane. Conformational dynamics underlie this process, allowing the transporter to sample at least two conformations, nominally grouped into inward- and outward-facing states. While studies of structure and dynamics have revealed atomistic insight into transport mechanisms and transport rates, the relative free energy differences among conformations remain underexplored. In this work, we quantified free energy differences between inward- and outward-facing conformations of the multidrug *E. coli* transporter EmrE using ^19^F NMR spectroscopy. EmrE consists of an antiparallel and asymmetric homodimer, where its quaternary structure resembles the inverted repeat structures found in larger nonoligomeric transporters. NMR experiments were performed using a minimal heterodimer of EmrE, where a single mutation was introduced into one monomer of the native EmrE homodimer. We discovered that a single conservative mutation perturbed the conformational equilibrium between inward- and outward-facing states by up to 1.5 kcal/mol. Surprisingly, we also found that as little as a single fluorine atom influenced the equilibrium by up to 0.8 kcal/mol. These measurements provide quantitative evidence that subtle changes influence the free energy landscape of a transporter, suggesting a plasticity that may be beneficial in divergent evolution to tune the transport mechanism.

## Graphical abstract

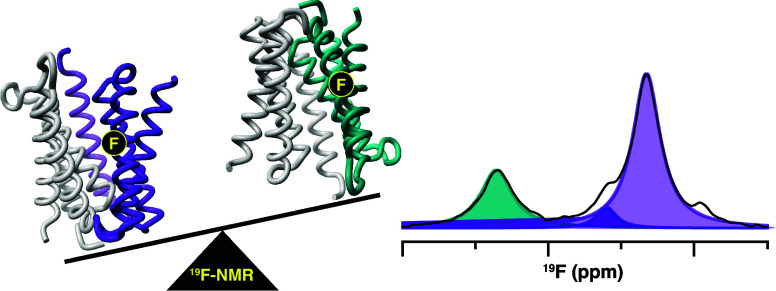

## Introduction

Membrane transport proteins in prokaryotes and eukaryotes translocate ions, amino acids, lipids, sugars, and drugs across the membrane. Active transport mechanisms are classified as primary or secondary, which harness ATP hydrolysis or an ion gradient as the energy source, respectively. Secondary transport, as proposed by Mitchell’s chemiosmotic hypothesis in 1961, uses electrochemical gradients, such as H^+^ or Na^+^, as the driving force for substrate transport against its concentration.^1,2^ Mitchell conceptualized a “mobile barrier” hypothesis to explain how transporters mediate substrate translocation across the membrane and how substrate binding modulates conformational changes of the protein. This hypothesis proposed more than 50 years ago remains consistent with the basic principles of alternating access.^3^ The foundation of this mechanism is that the substrate binding site is alternatively exposed to one side of the membrane at a time. High-resolution 3D structures and other biophysical insights have revealed three types of alternating access mechanisms: (i) rocker-switch, (ii) rocking-bundle, and (iii) elevator.^4–6^ The first two models operate by a mobile barrier, as postulated by Mitchell, while the third involves the sliding of one domain in the direction of the membrane bilayer with the other domain remaining fixed, and is termed a fixed-barrier mechanism.^7,8^ While structures in different conformations have been resolved and are essential for deciphering transport mechanisms, knowledge about relative free energies between states and the role of mutations in modulating the conformational energy landscape remains scarce.

Large transporters often contain two or more inverted repeat domains with low sequence homology,^9,10^ suggesting an evolutionary pathway through gene duplication and fusion of smaller ancestral transporter genes.^11,12^ The prevalence of pseudosymmetry among secondary transporters may contribute to substrate specificity and facilitate the alternating-access mechanism.^13^ Inverted repeats may also provide a means for reducing the free energy between inward- and outward-facing conformations.^9^ A potential progenitor family sharing the inverted repeat topology are transport proteins from the Small Multidrug Resistance (SMR) family. These 4-TM domain proteins^14,15^ have been a model system for studying membrane protein evolution since they assemble into antiparallel homodimers or heterodimers, which resemble the inverted repeat structure in larger transporters.^12,16,17^ SMR transporters are differentiated by the presence of a single gene producing a transporter that inserts in the membrane in both orientations (dual topology) or two genes producing transporters where each monomer inserts in a single but opposite orientation (single topology). The evolutionary advantage of single or dual topology proteins in the SMR family is poorly understood. Yet, it is likely that single topology genes arose through gene duplication events of dual topology genes.^12,18^ This aspect underscores the value of the SMR family as a model system for studying membrane protein transporter evolution.

In this work, we explored free energy differences between inward- and outward-facing conformations of the dual topology SMR transporter EmrE using ^19^F NMR spectroscopy. While energy landscapes have been studied with computational methods,^19–22^ experimental measurements of free energy changes remain a hurdle for understanding the catalytic cycles of transporters. By harnessing the power of ^19^F NMR spectroscopy, we quantified the free energy difference between inward- and outward-facing conformations using single mutations within the EmrE dimer to perturb the equilibrium. Our experimental results on these minimal heterodimers have implications for delineating fitness advantages involving divergent evolution for membrane proteins.

## Results and Discussion

### Conformational Bias Induced by a Single Fluorine Atom

^19^F NMR spectroscopy offers excellent signal-to-noise, is sensitive to the chemical environment, and has low abundance in native biomolecules, affording one-dimensional experiments and accurate quantification of populations.^23–31^ In particular, aromatic fluorine chemical shifts, such as those arising from 5′-fluoro-tryptophan, are hypersensitive due to the lone pair of electrons overlapping with adjacent π orbitals and serve as reporters for electrostatic changes.^32,33^

To investigate the free energy differences of EmrE, we targeted Trp63 within the protein for labeling with fluorine at the 5′ position of the indole ring (i.e., 5FW). Trp63 residues are centrally located in the substrate binding pocket of the EmrE dimer and close to the Glu14 residues (Figure 1a,b). Glu14 residues are essential for EmrE function since they bind protons in the ion-coupled transport mechanism. To enable unambiguous insight into EmrE’s energy landscape through the lens of Trp63, the other three tryptophan residues were mutated to phenylalanine (W31F, W45F, W76F). This mutant, herein referred to as EmrE^W63^, conferred ethidium bromide, proflavine, and methyl viologen resistance to *E. coli* to a similar extent as wild-type EmrE (Figures 1c and S1). EmrE^W63^ was also able to efflux ethidium, albeit with a reduced rate relative to wild-type EmrE (Figure S2).

**Figure 1:**
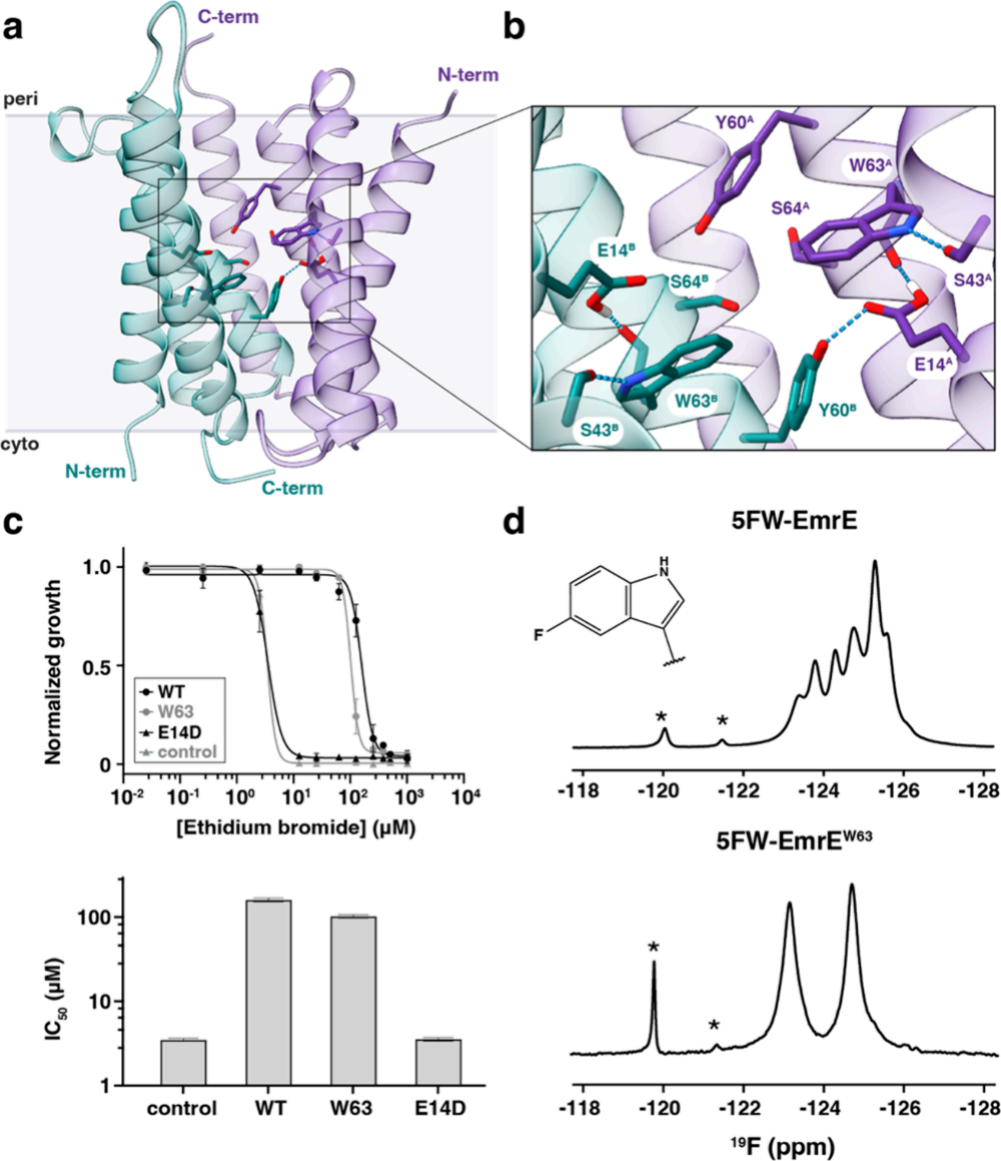
^19^F NMR spectroscopy is a sensitive reporter of the substrate binding pocket of EmrE. (a, b) Structure of proton-bound EmrE (PDB ID: 8UWU) displaying monomer A in purple, monomer B in teal, and select residues in the substrate binding site in sticks. Panel b displays an expanded view of panel a, where residues are displayed in a stick representation with the monomer chain indicated in the superscript. Dotted lines indicate hydrogen bonds for residues close to Trp63. (c) Top: growth inhibition experiments against ethidium bromide in liquid culture of *E. coli* ^Δ*acrB*Δ*emrE*Δ*mdfA*^ ^79^ expressing wild-type EmrE (WT), “W63” corresponding to the EmrE mutant W31F/W45F/W76F, or the E14D dead mutant of EmrE. Cells transformed with a plasmid without the ribosome binding site were used as the control. Error bars represent the standard deviation of measurements performed in triplicate. Relative growth was obtained by comparing OD_600 nm_ at 12 h of growth at 37 °C against the corresponding growth without ethidium bromide. Bottom: inhibition concentration at 50% (IC_50_) obtained from fitting the curves in the top panel, encompassing at least two independent experiments. Error bars represent the confidence interval at 95% from data fitting. IC_50_ values for WT (159.6 μM) and EmrE^W63^ (102.0 μM) are statistically different from the control and E14D mutant (*P*-value <0.0001). (d) One-dimensional ^19^F spectra of 5FW-labeled EmrE in 14:0-PC/6:0-PC isotropic bicelles at pH 5.6. 5FW-labeled wild-type EmrE and EmrE^W63^ are displayed in the top and bottom panels, respectively.

The one-dimensional ^19^F-NMR spectrum of 5FW-EmrE^W63^, obtained at pH 5.6, displayed two peaks of equal intensity and line width at −123.2 and −124.8 ppm (Figure 1d, bottom). At this pH value, the Glu14 residues are protonated; thus, the structure corresponds to the proton-bound structure previously published by our group,^34^ where the OH groups of Glu14 form hydrogen bonds with the backbone carbonyls of Trp63 (Figure 1b). The equal intensities in the fluorinated dimer indicated that the two monomeric species had equal populations and a relative free energy difference of zero, which is expected of a homodimer of EmrE. The resolved signals are referred to as A_2F_ and B_2F_ for monomers A and B, where the "2F" subscript corresponds to a dimer with two fluorine atoms. The separation of the peaks underscored the asymmetric and antiparallel structure of EmrE.^34–36^ Notably, these two signals in EmrE^W63^ matched peak positions in the wild-type EmrE ^19^F spectrum, albeit with overlapping peaks in the wild-type’s spectrum that obscured the chemical shifts of Trp63 (Figure 1d, top). To demonstrate that the two peaks matched those previously assigned to the proton-bound structure, we recorded ^19^F one-dimensional and ^1^H/^13^C HMQC two-dimensional spectra from heterodimers where one monomer was ^19^F-labeled and the other was ^13^C-labeled (Figure S3). These spectra matched those previously reported and confirmed the assignments.^34^

In addition to the two major signals in the one-dimensional ^19^F NMR spectrum of EmrE^W63^, we observed a minor peak with a chemical shift at −125.4 ppm (Figure 2a), which increased in intensity for samples with reduced ^19^F incorporation. To determine the identity of this signal, we mixed 5FW-EmrE^W63^ with natural abundance EmrE^W63^ at different molar ratios (Figure 2a). The intensity of the signal at −125.4 ppm increased with a greater fraction of natural abundance EmrE^W63^ present, while the intensity of A_2F_ decreased. This observation indicated the −125.4 ppm peak stemmed from monomer A in the dimer, where only this monomer had 5FW at Trp63. This signal is referred to as A_1F_. The assignment was validated using two-dimensional ^19^F/^19^F ZZ-exchange experiments of partially labeled EmrE^W63^ (Figure 2b). These spectra displayed correlations between the peak corresponding to monomer B with both A_1F_ and A_2F_, emblematic of conformational exchange.

**Figure 2:**
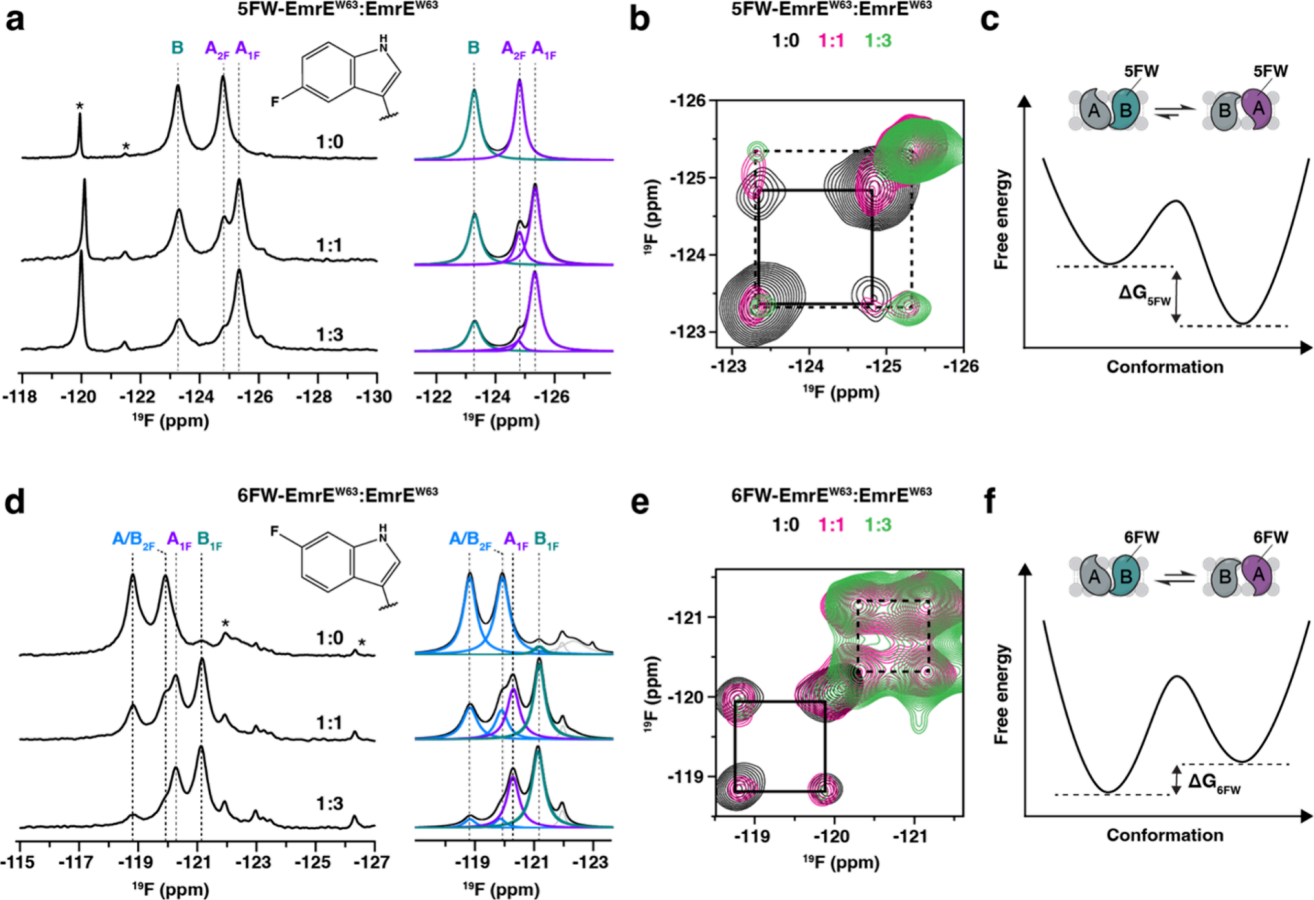
Conformational bias induced by a single fluorine atom. (a, d) One-dimensional ^19^F NMR spectra of (a) 5FW and (d) 6FW-labeled EmrE^W63^ (left panels) and the corresponding spectral deconvolution (right panels). Deconvoluted spectra are colored as follows: B in teal, A_1F_ or A_2F_ in purple, and unassigned A_2F_ or B_2F_ in blue. The summation of deconvoluted signals is displayed in black. The molar ratios of 5FW-EmrE^W63^ or 6FW-EmrE^W63^ to unlabeled EmrE^W63^ are displayed above each ^19^F spectrum. Asterisks indicate fluorine contaminants derived from the lipids used to prepare the bicelles. (b, e) Two-dimensional ^19^F/^19^F correlation spectra displaying overlays of (b) 5FW-EmrE^W63^ and (e) 6FW-EmrE^W63^ diluted with unlabeled EmrE^W63^. Spectra are colored according to the molar ratio of labeled to unlabeled protein (1:0 is black, 1:1 is magenta, and 1:3 is green). Spectra were obtained using a ZZ-exchange mixing time of 300 ms for the diluted samples, 150 ms for 5FW fully labeled EmrE, and 200 ms for 6FW fully labeled EmrE. Solid and dashed boxes indicate exchange between monomer A and monomer B within dimers containing two fluorines or one fluorine, respectively. (c, f) Summary of the impact on fluorine incorporation on EmrE’s conformational free energy landscape (5FW in panel (c) and 6FW in panel (f)). A single 5FW at residue 63 favors monomer A conformation in the EmrE dimer (0.8 kcal/mol), while a single 6FW has a slight preference for monomer B (0.2 kcal/mol).

A second observation from the 5FW-EmrE^W63^ and EmrE^W63^ sample mixed at a 1:3 molar ratio was that the A_1F_ signal displayed a greater integrated peak area relative to the monomer B signal (Figure 2a). This suggested the fluorine-labeled EmrE monomer preferred to occupy monomer A in EmrE, an effect arising from a single hydrogen to fluorine replacement in the dimer. Deconvolution of one-dimensional ^19^F NMR spectra and consideration of statistical mixing of the samples facilitated the calculation of the free energy, Δ*G*_5FW_ = 0.8 ± 0.2 kcal/mol, which stemmed from the single atom substitution (Figure 2c**;** see Methods for the calculation and Table S1). Remarkably, this observation indicated how a single atom substitution influenced the conformational equilibrium.

To test if fluorine labeling at a different position in the indole ring of Trp63 could induce a similar effect, we prepared a 6-fluoro-tryptophan labeled EmrE (6FW-EmrE^W63^) sample and assigned the signals in the spectrum. One-dimensional ^19^F NMR and two-dimensional ^19^F/^19^F NOESY spectra were acquired at different molar ratios with natural abundance EmrE^W63^ (Figure 2d,e), with peak assignments displayed in Figure S4. In contrast to 5FW-EmrE^W63^, monomer B_1F_ in 6FW-EmrE^W63^ displayed a slightly higher intensity compared to A_1F_. Deconvolution of these spectra enabled the free energy to be calculated, Δ*G*_6FW_ = −0.2 ± 0.1 kcal/mol. The negative sign indicates that the equilibrium favors the conformation where the fluorine-containing subunit is positioned in monomer B of the dimer (Figure 2f).

Overall, we found that the 5FW labeled monomer of EmrE preferred conformation A when paired with unlabeled EmrE, while the 6FW labeled monomer had a slight preference for conformation B. These findings indicate that the substitution of a single hydrogen atom with fluorine in a homodimer (5FW-EmrE^W63^ or 6FW-EmrE^W63^) disrupted the conformational equilibrium of the EmrE homodimer corresponding to alternating access exchange. Such a measurable effect on the equilibrium provides evidence for how minor perturbations within a protein, in this case a single atom, can influence the free energy difference between states. We hypothesize this observation supports a plasticity of the conformational free energy landscape, where mutational divergence influences the inward-facing to outward-facing equilibrium.

Lastly, we performed preliminary experiments to investigate substrate binding following addition of ethidium bromide to a heterodimer of 5FW-EmrE^W63^:EmrE (1:3) sample (Figure S5). Our spectra displayed reduced peak intensities upon substrate addition, suggesting faster conformational exchange and/or heterogeneity introduced upon ethidium binding. While we did not observe significant changes in Δ*G*, further experiments are needed to characterize the effect of substrates since EmrE binding is pH dependent.^34^

### Mechanism of Fluorine Bias Probed by MD Simulations

The central position of Trp63 in the binding pocket of EmrE facilitates interactions with hydrogen-bond donating residues, including Ser43 and Ser64 (Figure 1b). Since the interactions of Trp63 in monomer A and B are not identical, stemming from the asymmetric dimer structure, we hypothesized that differential contacts with fluorine-labeled derivatives of Trp63 were responsible for the observed conformational preference in the 5FW-EmrE^W63^ heterodimer with EmrE^W63^. Participation of fluorine atoms in hydrogen bonds has been recognized in proteins and in protein-drug interactions.^37,38^

To test this hypothesis, we performed MD simulations starting from the proton-bound EmrE structure (PDB ID: 8UWU) in explicit DMPC lipid bilayers following introduction of fluorine at the 5′ or 6′ indole position of Trp63 in each monomer (see details in Table S2). All-atom MD simulations were run for a total of ∼1.65 μs, which enabled local conformational changes to be analyzed within the substrate binding pocket. Notably, this time scale was insufficient for probing larger conformational changes associated with alternating access exchange since these dynamics occur on a msec to sec time scale.^35,39,40^ The simulation results revealed that 5FW-EmrE made one extra hydrogen bond when the fluorine atom was positioned in monomer A relative to monomer B (Figure 3a,b), without inducing any significant changes in the overall structure of EmrE and the side chain rotamer of Trp63 (Figure S6a,b). Further analysis of these interactions indicated no major differences in intramolecular contacts, yet a significant increase in the number of intermolecular contacts of 5FW in monomer A with Tyr60 and Ser64 in monomer B (Figure S6c–e). These results agree with the more shielded monomer A ^19^F chemical shift, as the formation of hydrogen bonds typically shields the fluorine atom.^41^ In contrast, simulations on 6FW-EmrE made a similar number of total hydrogen bond contacts when 6FW was introduced in monomer A or B (Figure 3c,d). However, monomer B displayed a higher number of intramolecular contacts relative to monomer A, which may explain the slight preference for 6FW to occupy monomer B in the NMR experiments. Overall, these results support a mechanism underlying the fluorine bias detected using ^19^F NMR spectroscopy where the location of the introduced fluorine atom differentially contributes to hydrogen bonding in each monomer of the asymmetric dimer.

**Figure 3:**
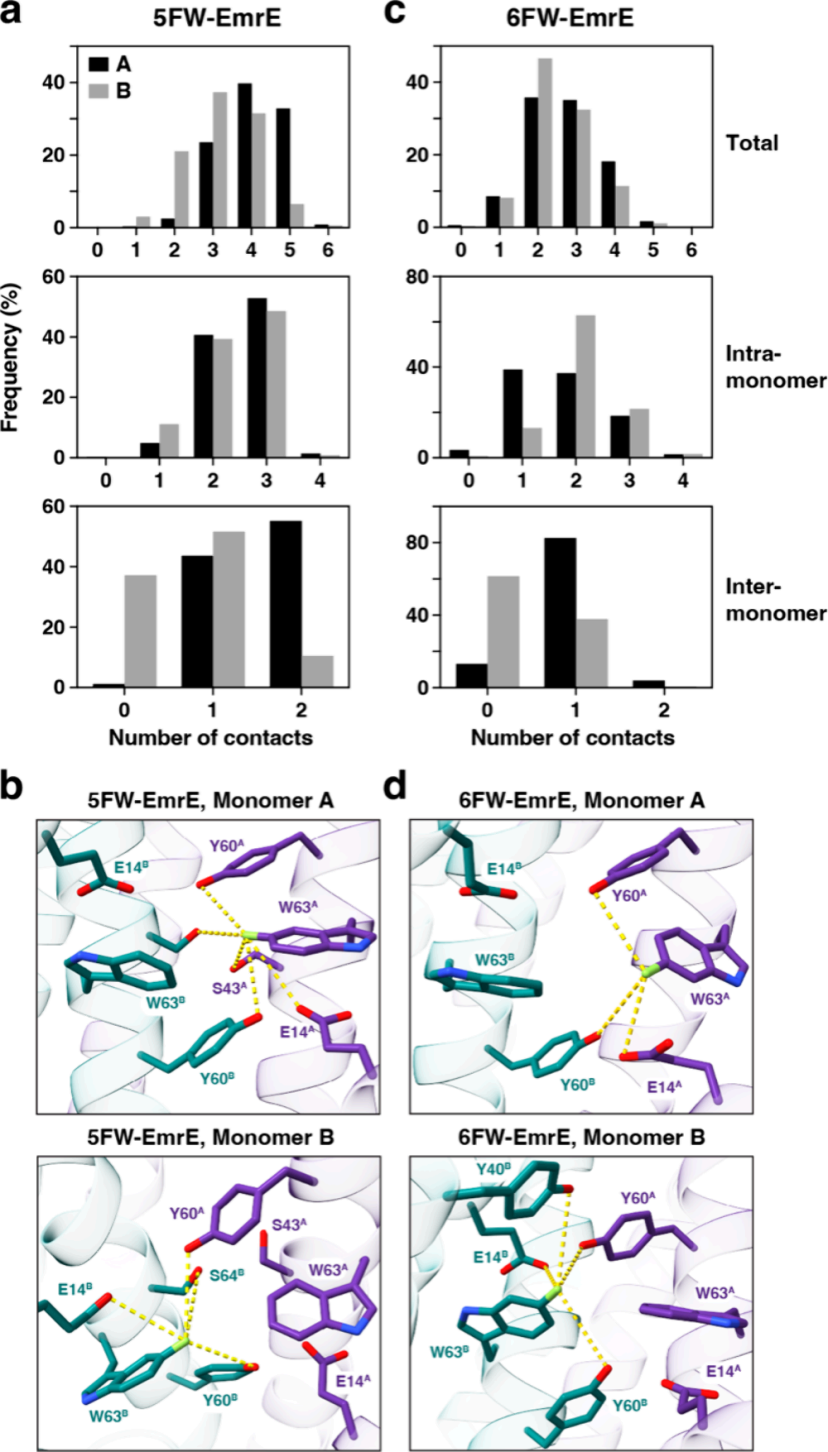
Mechanism of fluorine bias probed by MD simulations. (a, c) Frequency of hydrogen bond formation (≤5 Å between heavy atoms) quantified from MD simulations where a fluorine atom was placed in monomer A or B of (a) 5FW-EmrE or (c) 6FW-EmrE. Total hydrogen bond contacts are displayed as the total number of contacts (top row), intramonomer contacts (middle row), and intermonomer contacts (bottom row). Black and gray bars indicate monomer A and monomer B, respectively. (b, d) Representative snapshots from MD simulations performed on (b) 5FW-EmrE and (d) 6FW-EmrE with the fluorine atoms introduced as labeled in light green. Dashed yellow lines indicate hydrogen bonds ≤5 Å observed during the simulations.

### Free Energy Quantification for Single Mutations in the EmrE Dimer

The influence of the conformational equilibrium by a single fluorine atom suggested that the conformational balance of EmrE can also be influenced by relatively minor mutations. The separation and homogeneity of the two Trp63 signals for the proton-bound state of EmrE would enable us to quantitatively assess the free energy of the conformational equilibrium due to these single mutations within the dimer. Namely, while an EmrE homodimer displayed a 50/50 population of conformations A and B, i.e., equal energy in both outward- and inward-open conformations, altering this distribution would offer a quantitative measure of mutant-induced perturbation. Such a measurement would resemble a subfamily of transporters within the SMR family requiring the expression of two single topology genes to pair together for function. As proof of principle, our experiments focused on EmrE^W63^ mixed with the corresponding L51I or E14Q mutants (EmrE^W63, L51I^ or EmrE^W63, E14Q^), where only one of the two proteins was fluorinated.

First, we prepared a mixed sample of 5FW-EmrE^W63, L51I^ and EmrE^W63^ at a molar ratio 1:3 and collected a one-dimensional ^19^F NMR spectrum. This experiment displayed a prominent peak at −125.1 ppm assigned to monomer A and a smaller signal at −123.3 ppm assigned to monomer B (Figure 4a). This result revealed a conformational preference for the L51I mutation to assume the monomer A position in the heterodimer. Likewise, a sample with swapped labeling, 5FW-EmrE^W63^ mixed with natural abundance EmrE^W63, L51I^, displayed a major peak at −123.3 ppm, assigned to monomer B, and two minor ones at −124.8 and −125.4 ppm (Figure 4b). The latter two signals arise from monomer A in a homodimer with two fluorine atoms or monomer A in a heterodimer with one fluorine, as described in the prior section. To calculate the free energy difference, spectral deconvolution was performed (Figure 4a,b, right panels). The apparent free energy differences (Δ*G*_app_) for 5FW-EmrE^W63, L51I^:EmrE^W63^ and EmrE^W63, L51I^:5FW-EmrE^W63^ samples were 2.3 and 0.7 kcal/mol, respectively. Notably, these free energy differences were produced by two factors, the effects of the L51I mutation and the effects of 5FW-labeling. The effect of 5FW, calculated at Δ*G*_5FW_ = 0.8 kcal/mol in the previous section, was additive for the 5FW-EmrE^W63, L51I^:EmrE^W63^ sample since the mutation and labeling were in the same monomer and each produced a preference for monomer A. Oppositely, the observed free energy difference of the 5FW-EmrE^W63^:EmrE^W63, L51I^ sample was the difference between the effect of the 5FW labeling and the effect of the mutation L51I since these two features were in different monomers in the heterodimer. Averaging the results of the two samples, the free energy associated with the mutation L51I was Δ*G*_L51I_ = 1.5 ± 0.1 kcal/mol (Table 1).

**Figure 4:**
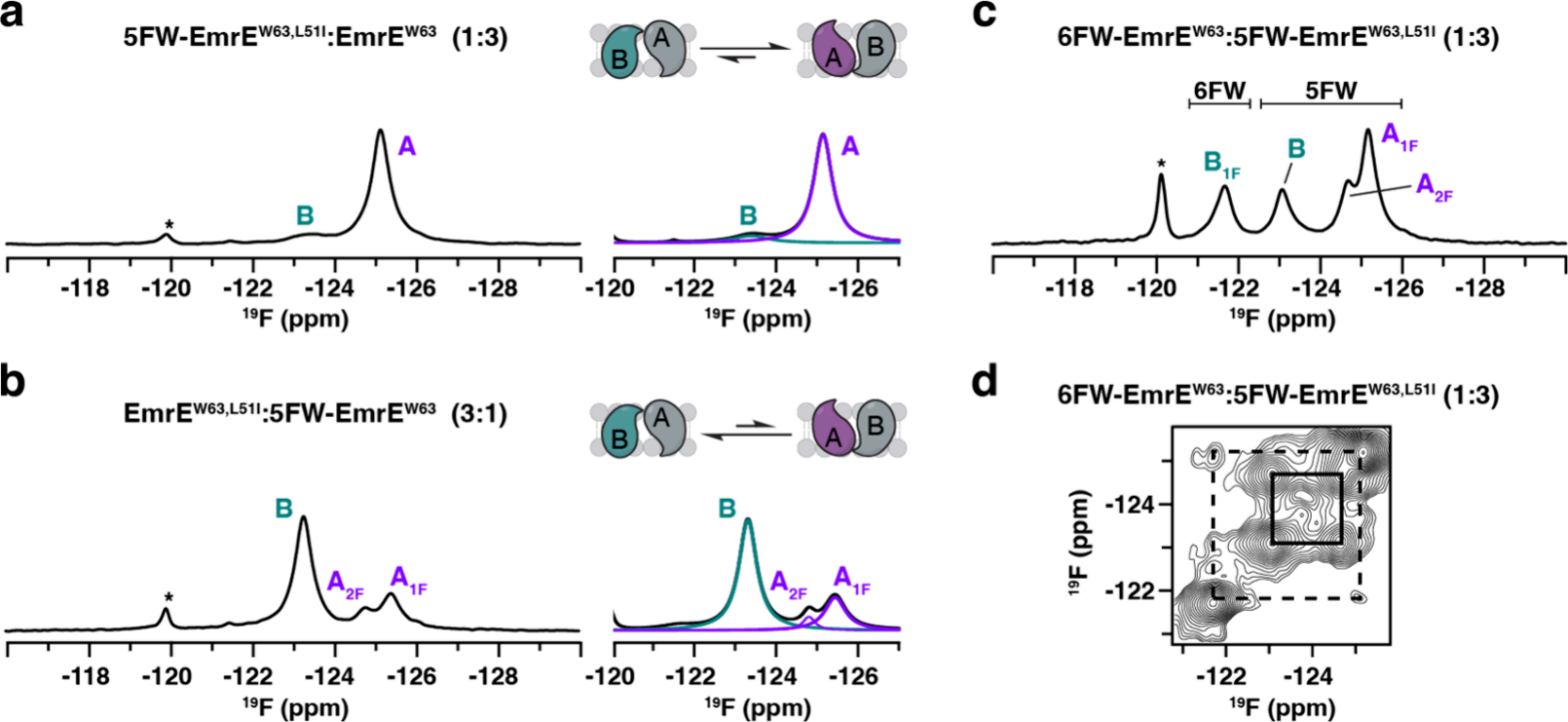
^19^F NMR measurements to determine the free energy associated with the single L51I mutation in the EmrE dimer. (a, b) One-dimensional ^19^F NMR spectra of (a) 5FW-EmrE^W63, L51I^:EmrE^W63^ at a 1:3 molar ratio and (b) EmrE^W63, L51I^:5FW-EmrE^W63^ at a 3:1 molar ratio (left panels) and the corresponding spectral deconvolution (right panels). Deconvoluted spectra are colored as follows: B in teal and A_1F_ or A_2F_ in purple. The summation of deconvoluted signals is displayed in black. (c) One-dimensional ^19^F NMR spectrum of the 5FW-EmrE^W63,L51I^:6FW-EmrE^W63^ heterodimer formed with a 3-fold excess of the 5FW-labeled protein. Monomer peak assignments are colored similarly to panels (a) and (b), with the 5F and 6F peaks indicated above the spectrum. (d) Two-dimensional ^19^F/^19^F correlation spectra of the 5FW-EmrE^W63, L51I^:6FW-EmrE^W63^ heterodimer formed with a 3-fold excess of the 5FW-labeled protein. Solid and dotted boxes indicate cross-peaks between monomers A and B of the 5FW-EmrE^W63, L51I^ homodimer (i.e., two fluorines in dimer) and an NOE signal between monomers A and B of the 5FW-EmrE^W63, L51I^:6FW-EmrE^W63^ heterodimer, respectively.

**Table 1:** Free Energy of the Samples Measured in This Work

**sample**	**equilibrium** ^a^	**Δ*G***_**apparent**_ (kcal/mol)	**Δ*G*** ^b^ (kcal/mol)
5FW-EmrE^W63^:EmrE^W63^	${\underset{-}{A}}^{W63}\cdot B^{W63}⇌{\underset{-}{B}}^{W63}\cdot A^{W63}$		0.8 ± 0.2 (*n* = 6)
6FW-EmrE^W63^:EmrE^W63^	${\underset{-}{A}}^{W63}\cdot B^{W63}⇌{\underset{-}{B}}^{W63}\cdot A^{W63}$		–0.2 ± 0.1 (*n* = 2)
5FW-EmrE^W63, L51I^:EmrE^W63^	${\underset{-}{A}}^{W63,L51I}\cdot B^{W63}⇌{\underset{-}{B}}^{W63,L51I}\cdot A^{W63}$	2.3 ± 0.1 (*n* = 2)	1.5 ± 0.2
EmrE^W63, L51I^:5FW-EmrE^W63^	$A^{W53,L51I}\cdot {\underset{-}{B}}^{W63}⇌B^{W63,L51I}\cdot {\underset{-}{A}}^{W63}$	0.76 ± 0.05 (*n* = 3)	1.6 ± 0.2
EmrE^W63^:5FW-EmrE^W63, E14Q^	$A^{W63}\cdot {\underset{-}{B}}^{W63,E14Q}⇌B^{W63}\cdot {\underset{-}{A}}^{W63,E14Q}$	0.47 ± 0.05 (*n* = 3)	1.3 ± 0.2
5FW-EmrE^W63^:EmrE^W63, E14Q^	${\underset{-}{A}}^{W63}\cdot B^{\mathrm{WQ}63,E14}⇌{\underset{-}{B}}^{W63}\cdot A^{W63,E14Q}$	1.5 (*n* = 1)	0.7

a Underlined monomer A or B indicates the location of the fluorine label.

b Errors displayed without an *n* value in parentheses reflect error propagation. The *n* value corresponds to the number of independent experiments performed.

We envisioned that the favorable 5FW position and L51I mutation for monomer A and 6FW position for monomer B could be combined to yield an even greater conformational equilibrium change. As such, we mixed 6FW-EmrE^W63^ with a 3-fold molar excess of 5FW-EmrE^W63, L51I^ and collected ^19^F NMR experiments (Figure 4 **c, d**). Only the peak corresponding to monomer B_1F_ was observed in the 6FW region (Figure 4c), in agreement with a greater conformational bias expected from the additive effect of each fluorine position.

Next, we analyzed the effect of the E14Q mutation on the conformational equilibrium when this mutant was paired with EmrE^W63^. These experiments were designed to investigate the similarity of glutamic acid and glutamine, where glutamine is the closest mimic of a protonated carboxylic acid. In our NMR structure of EmrE (PDB ID 8UWU), the protonated Glu14 side chain forms a hydrogen bond with the backbone carbonyl of Trp63. Therefore, changing the OH group of glutamic acid to NH_2_ in glutamine would introduce a geometry difference and a change in hydrogen bond donor strength. The latter stems from oxygen being more electronegative than nitrogen.

^19^F NMR spectra of the EmrE^W63, E14Q^ homodimer and EmrE^W63^:EmrE^W63, E14Q^ heterodimers were acquired at pH 5.6 (Figure 5 **a-c**). The first notable observation was that the 5-fluoro Trp63 peaks in the EmrE^W63, E14Q^ homodimer were shifted by 1–2 ppm compared to 5FW-EmrE^W63^ (Figure 5a), which was expected given the proximity (<5 Å) between the fluorine atom of 5FW-Trp63 to Glu14 in the proton-bound EmrE structure (Figure 1b). The second key finding was that heterodimer spectra comprised of 5FW-EmrE^W63, E14Q^ mixed with EmrE^W63^ displayed the presence of a new signal at −122 ppm that corresponded to monomer B with one fluorine (B_1F_) (Figure 5a). This signal increased in intensity for the 1:3 molar ratio sample relative to the 1:1 sample, indicating that the Gln14 mutant monomer preferred position B in a heterodimer with EmrE^W63^. Likewise, the opposite labeling scheme of 5FW-EmrE^W63^ mixed with natural abundance EmrE^W63, E14Q^ displayed a major peak corresponding to monomer A (A_1F_) (Figure 5b) with the peak in a similar position as in Figure 2a. This indicated that when the E14Q mutation was in the opposing monomer relative to the 5FW labeling, there was no significant chemical shift perturbation in monomer A. These observations indicated that the Gln14 mutation preferred monomer B, supporting the conclusion that different conformational preferences existed for these two similar residues.

**Figure 5:**
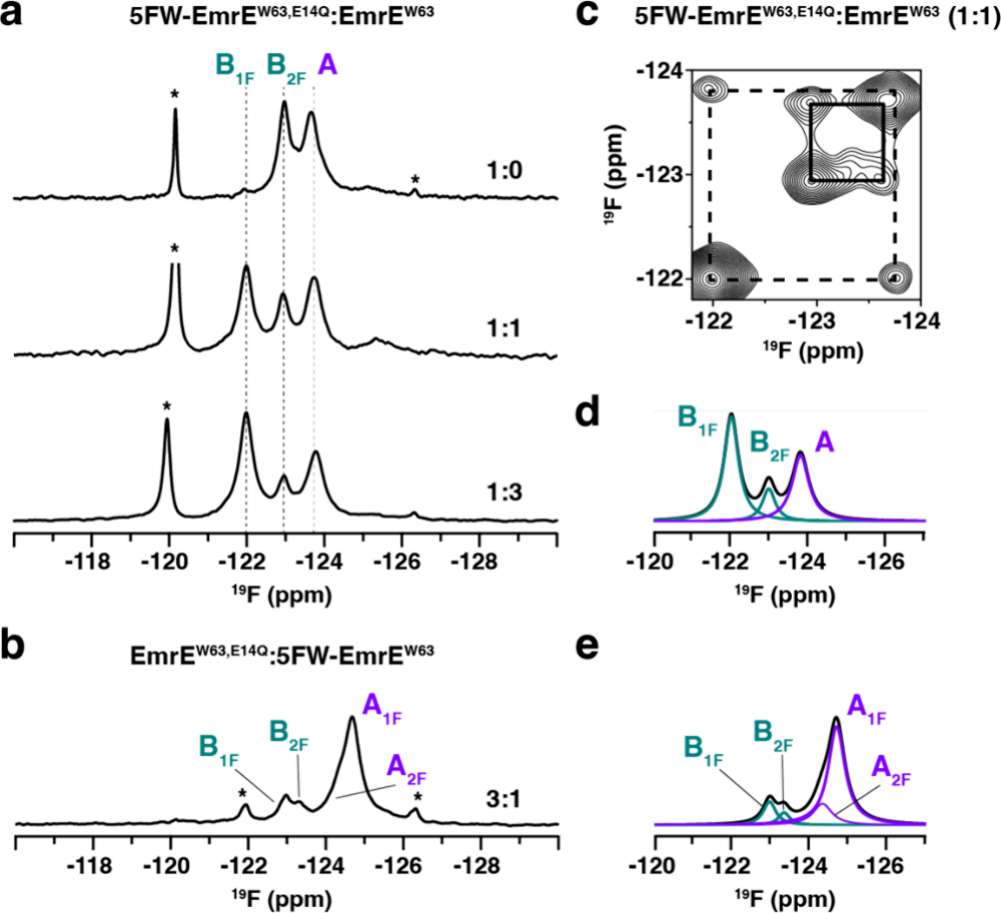
^19^F NMR measurements to determine the free energy associated with the E14Q mutation in the EmrE dimer. (a) One-dimensional ^19^F NMR spectra of the (a) 5FW-EmrE^W63, E14Q^ homodimer (top) and the 5FW-EmrE^W63, E14Q^:EmrE^W63^ heterodimer prepared at a molar ratio of 1:1 (middle) or 1:3 (bottom). (b) One-dimensional ^19^F NMR spectrum acquired on a EmrE^W63, E14Q^:5FW-EmrE^W63^ heterodimer sample formed by mixing the proteins in a 3:1 molar ratio. (c) Two-dimensional ^19^F/^19^F correlation spectra of the 5FW-EmrE^W63, E14Q^:EmrE^W63^ heterodimer sample prepared by mixing the proteins at 1:1 molar ratio. Solid and dotted boxes indicate the correlations between monomers in the homodimer and heterodimer, respectively. (d, e) Deconvolutions of the EmrE^W63, E14Q^:EmrE^W63^ heterodimer spectra on the left of panels a (bottom) and b. Deconvoluted spectra are colored as follows: monomer A in purple and monomer B in teal. The summation of deconvoluted signals is displayed in black.

Deconvolution of spectra in Figure 5a,b allowed for quantification of populations of monomers A_1F_ and B_1F_ in the EmrE^W63^:EmrE^W63, E14Q^ heterodimer samples (Figure 5d,e). These calculations yielded Δ*G*_app_ in 5FW-EmrE^W63, E14Q^:EmrE^W63^ and EmrE^W63, E14Q^:5FW-EmrE^W63^ heterodimers equal to −0.47 ± 0.05 and −1.5 kcal/mol, respectively, where the negative sign corresponds to a free energy with the E14Q mutant favoring monomer B in the heterodimer. Due to the preference of 5-fluoro Trp63 to occupy monomer A, these Δ*G*_app_ values needed to be corrected by Δ*G*_5FW_ = 0.8 ± 0.2 kcal/mol to isolate the influence of the E14Q mutation. The resulting free energies arising from the E14Q mutation (Δ*G*_E14Q_) in the 5FW-EmrE^W63, E14Q^:EmrE^W63^ and EmrE^W63, E14Q^:5FW-EmrE^W63^ samples were −1.3 ± 0.1 and −0.7 kcal/mol, respectively (Table 1). However, due to the proximity of 5-fluoro Trp63 and Gln14 in the same monomer (Figure 1b), likely giving rise to an additional interaction, we postulated that the former free energy was less accurate of the glutamine mutant’s influence on the conformational preference. Thus, the value of −0.7 kcal/mol obtained from the heterodimer EmrE^W63, E14Q^:5FW-EmrE^W63^ is more likely to represent the free energy effect of only the E14Q mutation when in a heterodimer with EmrE possessing the native Glu14 residue in the protonated state. Taken together, these results suggest that the chemical environments of the two Glu14 residues are different, leading the Gln14 mutant to favor the monomer B conformation when mixed with EmrE containing the native Glu14 residue. The sensitivity of these ^19^F NMR experiments to small free energy changes in the EmrE conformational equilibrium will likely be valuable for studying the energy landscape of paired SMR subfamily transporters comprised of two oppositely oriented proteins.

### Discussion of Evolutionary Implications

According to Darwin’s theory, complex systems evolved from simpler ones through “*numerous, successive, slight modifications*” under the influence of natural selection.^42^ Protein evolution can be treated similarly, where mutations, insertions/deletions, and recombination leading to minor changes, referred to as microtransitions, can lead to new functions.^43^ This is particularly pertinent for membrane proteins since they are thought to diverge more rapidly in their external-facing portions than intracellular soluble proteins due to stronger selective pressure of changing environments.^44–46^ This characteristic and others make membrane proteins less conserved and more difficult to study with phylogenetic methods. A complementary approach is to quantify how protein function changes from mutations by considering the free energy landscape,^47^ which represents the relative energy distribution of all possible protein conformations.^48–50^ Our findings provide quantitative evidence of the free energy landscape induced by the simplest microtransition to a homodimer, a single conservative mutation and a single atom.

Many single-chain polypeptide secondary transporters in nature contain structural repeats relative to those relying on oligomer formation for function. This observation is consistent with Dayhoff’s hypothesis that large proteins emerged from shorter peptides or domains that duplicated, fused, and diversified to form novel functions.^51^ Numerous studies have demonstrated that gene duplication is a ubiquitous source of functional novelty^52–57^ that enhances protein evolvability and favorable functions.^58,59^ This suggests an evolutionary advantage of single polypeptide chain transporters versus oligomeric systems. One example is the drug/metabolite transporter (DMT) superfamily found in bacteria, archaea, and eukaryotes that is comprised of 44 families (Transporter Classification Database number 2.A.7), yet only a few are comprised of smaller progenitor genes that form oligomers.^60,61^ These progenitor families consist of four and five TM proteins found mainly in bacteria, e.g., the SMR and BAT families, compared to the 10 TM domain families found in all kingdoms of life^62–64^ and thought to be the product of intragene duplication of their primordial five TM domain proteins.^60^ Likewise, 12 TM domain transporters from the major facilitator superfamily (MFS), the largest family of secondary transporters,^65^ are thought to have evolved through duplication and fusion of smaller genes, possibly through two sequential duplication events of a three TM domain precursor.^66,67^

#### Why Might Evolution Favor Larger Transporter Structures with an Inverted Repeat Fold?

We hypothesize one reason is that a single gene can accommodate a single mutation, while a mutation to a gene forming an oligomeric protein would introduce multiple mutations in the functional assembly. A key caveat of this discussion is how two or more mutations in the homooligomer interact with each other; i.e., whether they produce cooperative or canceling effects.^68^ In the case of an additive or cooperative effect, there may be favorable functionality achieved, such as those involving stabilizing an oligomer or amplifying effects from allosteric mutations. However, anticooperativity of the mutations might produce a canceling effect relative to one mutation introduced into a single domain protein.

Our NMR experiments reveal that the most minor perturbations to one subunit of a homodimer, a single atom substitution or a conservative mutation to one residue, influenced the equilibrium between inward- and outward-facing conformations by ∼13-fold, corresponding to a free energy change of 1.5 kcal/mol. In contrast, the same mutation in both monomers of the homodimer led to a canceling effect with no change in the conformational free energy. These findings support the idea that one mutation in a heterodimer or a single polypeptide chain may serve an advantageous role in evolution. A schematic of this hypothesis is presented in Figure 6 as a plot of differentiation along the *y*-axis and genetic drift along the *x*-axis. The change in the equilibrium between inward- and outward-facing conformations is one example of what we term differentiation which is essential for secondary active transport. Additional differentiation factors for transporters pertain to other aspects of the transport mechanism, including proton binding and substrate selectivity.

**Figure 6:**
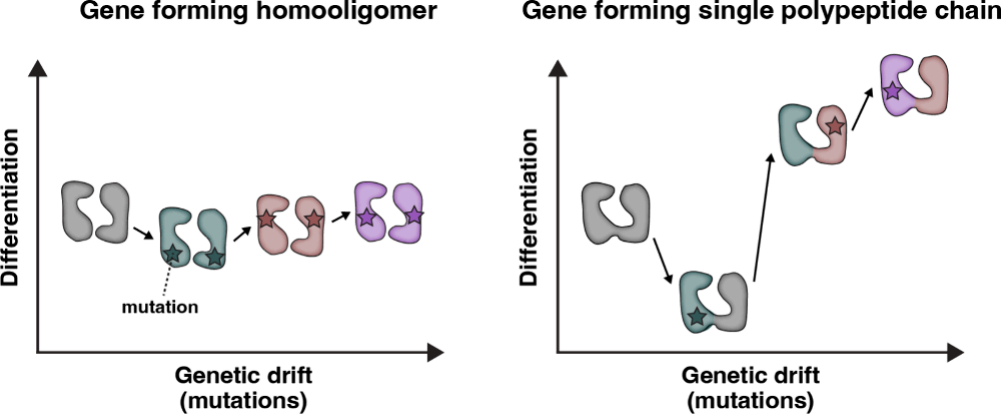
Hypothesis about evolutionary advantages of a single polypeptide chain. The schematic depicts differentiation of transport properties along the *y*-axis versus genetic drift along the *x*-axis, where mutations are denoted with stars. Each step, from left to right, introduces one mutation into the gene. For genes forming a homodimer, this results in two mutations in the homooligomer, while genes comprised of a single polypeptide chain result in one mutation. The case for homooligomers is one where mutations at each step are not additive or cooperative. The hypothesis graphically displayed in the figure is that mutations to a single polypeptide gene display a greater propensity for functional differentiation relative to smaller genes that form homooligomers. Note that differentiation refers to a property of a transporter that could impact a step in a transporter’s catalytic cycle, such as alteration of the inward- to outward-facing equilibrium and proton or substrate binding. Differentiation might increase or decrease the functional output of a transport cycle.

In conclusion, our minimalist heterodimeric experiments where a single residue or atom was introduced into the dimer show how minor changes quantitatively impact free energy differences of inward- and outward-facing conformations. The relatively large changes in free energies, up to 1.5 kcal/mol, support the hypothesis that minor changes occurring during mutational divergence may have a significant impact on conformational equilibria required for function. We hypothesize that this *dynamic free energy landscape* may have served as a driving force for selecting single-gene fusion transporters over homooligomers.

## Materials and Methods

### EmrE Expression and Purification of ^19^F-Labeled Proteins

An EmrE mutant with one tryptophan in the primary sequence (EmrE^W63^) was engineered by mutating three native tryptophan residues to phenylalanine (W31F/W45F/W76F). EmrE^W63^ was encoded as a fusion protein with maltose-binding protein into the pMal-c2x vector and expressed in *E. coli* BL21­(DE3). Natural abundance EmrE^W63^ was produced in regular LB medium.

To produce 5-fluoro-tryptophan or 6-fluoro-tryptophan labeled EmrE^W63^, cells were grown in LB medium up to OD_600 nm_ of ∼0.8, then the medium was exchanged to M9 minimal medium supplemented with 1 g/L of glyphosate (Shikimate pathway blocker), 60 mg/L of the corresponding fluoro-indole (fluoro-tryptophan precursor), and the other 19 amino acids at natural abundance. For natural abundance amino acids, 800 mg/L of tyrosine, phenylalanine, alanine, glycine, serine, and cysteine were added to the growth medium and 300 mg/L of all other amino acids. Cells were incubated in this medium at 37 °C for 1 h prior to IPTG induction.

EmrE was purified as previously reported by passing the bacterial lysate over an amylose column and cleaving with TEV protease.^69^ Next, EmrE or mutants were purified by size exclusion chromatography using a Superdex 75 10/300 GL column (Cytiva) in 20 mM phosphate buffer at pH 7.3, 150 mM NaCl, and 0.06% *n*-dodecyl-β-d-maltopyranoside (DDM) (w/v).

### NMR Samples Preparation

All solution NMR studies were performed in isotropic bicelles. Labeled EmrE^W63^ was reconstituted into 1,2-di-*O*-tetradecyl-*sn*-glycero-3-phosphocholine (14:0 PC) and 1,2-di-O-hexyl-*sn*-glycero-3-phosphocholine (6:0-PC) bicelles with a long-chain to short chain molar ratio 1/3 (*q* = 0.33). The heterodimer samples were prepared as follows. Immediately prior to lipid reconstitution, fluoro-tryptophan labeled EmrE^W63^ or mutant was mixed with its unlabeled partner at a molar ratio of 1:3 unless otherwise specified, keeping an excess of the NMR silent protein. The two mixed proteins were incubated in DDM for 1 h at 37 °C while vigorously stirring in the presence of 10 mM DTT. The total protein concentration in the reconstitution mixtures was 40 μM, and the protein-to-lipid ratio was 1:30 (mol:mol). After incubation at 37 °C, lipids were added, and the mixture was stirred for 1 h at room temperature. DDM was removed by the addition of Biobeads SM-2 resin (Bio-Rad) in 100-fold excess relative to DDM (w/w). Proteoliposomes were recovered by ultracentrifugation at 347,500*g* using a TLA-110 rotor (Beckman-Coulter) and dissolved with 6:0-PC prior to NMR measurements.

### ^19^F NMR Spectroscopy

One-dimensional ^19^F experiments were performed at protein concentrations between 1 and 2 mM in 150 mM Na_2_HPO_4_, 20 mM NaCl, containing 10% D_2_O (v/v). Each experiment was collected using an 800 MHz Bruker AVANCE NEO spectrometer equipped with a TCI cryogenic probe, which possesses a high-frequency channel tunable to ^19^F (753 MHz). Spectra were acquired at 37 °C with 5000 transients, a spectral width of 50 ppm, and a recycle delay of 1 s. The measurement time was approximately 90 min per experiment. ^19^F/^19^F NOESY were acquired with a mixing time of 150, 200, or 300 ms, 256 transients, 32 increments, and a recycle delay of 1 s. Spectral widths were 37,037 Hz and 4517 Hz in the direct and indirect dimensions, respectively.

### Free Energy Calculation

The equilibrium and equilibrium constant for a heterodimer formed by EmrE^W63^ and an EmrE^W63^ variant are displayed in eqs 1 and 2, respectively.

$${\mathrm{EmrE}}_{A}^{W63}\cdot \mathrm{FW}‐{\mathrm{EmrE}}_{B}^{W63,\mathrm{variant}}⇌\mathrm{FW}‐{\mathrm{EmrE}}_{A}^{W63,\mathrm{variant}}\cdot {\mathrm{EmrE}}_{B}^{W63}$$ 1

$$K=\frac{p_{B,\mathrm{het}}}{p_{A,\mathrm{het}}}=\frac{I_{B,\mathrm{het}}}{I_{A,\mathrm{het}}}$$ 2

FW is the location of the fluoro-tryptophan (5FW or 6FW), “variant” is a mutant of EmrE, subscripts indicate the EmrE monomer (A or B) corresponding to the chain in PDB ID 8UWU, *p*_A,het_ and *p*_B,het_ are the populations of the heterodimers, and *I*_A,het_ and *I*_B,het_ are the integrated intensities obtained from one-dimensional NMR experiments. Note that 5FW or 6FW can also be located on EmrE^W63^ when mixed with an EmrE mutant at natural abundance. Spectral deconvolutions and integrations were performed using Topspin 4.3.0.

Due to mixing used to prepare the heterodimers, samples contain a fraction of homodimers (*f*_homo_) and heterodimers (*f*_het_) that depend on the ratio of labeled and unlabeled species. For example, samples containing one part labeled protein to three parts unlabeled protein correspond to *f*_homo_ = 1/7 and *f*_het_ = 6/7, assuming statistical mixing. To obtain the equilibrium constant as in eq 2, integrations of heterodimer peaks corresponding to monomers A and B would ideally be sufficient for this calculation. However, the heterodimer peak positions overlapped with those of the homodimer and required the following modification of eq 2.

$$K=\frac{I_{B,\mathrm{homo}}-I_{A,\mathrm{homo}}+I_{B,\mathrm{het}}}{I_{A,\mathrm{het}}}=\frac{I_{B}-I_{A,\mathrm{homo}}}{I_{A,\mathrm{het}}}$$ 3

*I*_A,homo_ is the integral of monomer A in the homodimer, *I*_A,het_ is the integral of monomer A in the heterodimer, and *I*_B_ is the integral of monomer B for the homodimer and heterodimer. Equation 3 is appropriate since the populations and integrated intensities of homodimer are equal by definition; i.e., *p*_A,homo_ = *p*_B,homo_ and *I*_A,homo_ = *I*_B,homo_.

Equilibrium constants were calculated using eq 3, which were subsequently used to determine the Gibbs free energy (Δ*G*) from eq 4 by setting the temperature (*T*) equal to 310 K.

ΔG=RTlnK 4

The 5FW-EmrE^W63^:EmrE^W63^ experiments resulted in the free energy calculation due to fluorine (Δ*G*_FW_) directly from eqs 3 and 4. For determination of the equilibrium for the heterodimer EmrE^W63, L51I^:EmrE^W63^ and EmrE^W63, E14Q^:EmrE^W63^, we first determined the apparent equilibrium constants and corresponding Gibbs free energies Δ*G*_*app*_ from eqs 3 and 4, respectively. Next, we used eq 5 to determine the contribution of the mutation Δ*G*_*mut*_ after considering the fluoro-tryptophan free energy correction (Δ*G*_FW_).

$$\Delta G_{\mathrm{app}}=\Delta G_{\mathrm{mut}}+\Delta G_{\mathrm{FW}}$$ 5

### Molecular Dynamics Simulation of Fluorinated EmrE in DMPC Bilayers

The proton-bound state EmrE (PDB ID: 8UWU) was used to initiate MD simulations. Trp63 was modified to 5FW or 6FW in monomer A or B, and both Glu14 residues were protonated. Each structure was embedded into DMPC bilayers using the OPM server^70^ in CHARMM-GUI.^71^ Each system was set up following established models for membrane proteins including DMPC lipids,^72^ TIP3P molecules,^73^ and sodium chloride. The CHARMM36 force field implemented in GROMACS 2020.4 ^74^ was used with parametrization for the nonstandard amino acids 5-fluoro-tryptophan and 6-fluoro-tryptophan.^75^ Equilibration and all-atom MD simulations were performed following the reported procedure.^34^ Briefly, a steepest descent minimization was completed until the maximum force was below 1000 kJ mol^–1^ nm^–2^. Next, the system was equilibrated for 10 ns under constant volume temperature with heavy atoms of the protein constrained. From there, a constant pressure temperature simulation where heavy atoms of the protein (1000 kJ mol^–1^ nm^–2^) were gradually reduced to 0 kJ mol^–1^ nm^–2^ for a total of 30 ns following an additional 30 ns of unconstrained MD prior to production MD simulations. Temperature was maintained at 310.15 K using the Nose–Hoover thermostat.^76^ Pressure was maintained at 1 bar using the Parrinello–Rahman coupling.^77^ Coordinates were saved every 5 ps and analyzed using GROMACS 2020.4 and VMD.^78^ Each simulation was run for 550 ns starting from three random velocities for each fluorinated form of EmrE.

### Growth Inhibition Experiments in *E. coli*

Growth inhibition assays were performed like those previously described.^34^ In brief, *E. coli* ^Δ*acrB*Δ*emrE*Δ*mdfA*^ cells were transformed with the vector PMS119EH containing EmrE, EmrE^W63^, EmrE^E14D^, or EmrE without a ribosome binding site as a control. A single colony was grown for ∼20 h at 37 °C in TBG medium supplemented with 100 μg/mL carbenicillin. Next, cultures were diluted 300-fold into fresh TBG medium supplemented with 100 μg/mL carbenicillin and the compound to be tested. The optical density at 600 nm was measured every 15 min using a Bioscreen Pro C instrument at 37 °C with slow shaking. The OD_600 nm_ at 12 h (for ethidium) or 24 h (for methyl viologen and proflavine) were fit to the nonlinear “Sigmoidal, 4PL, X is concentration” equation using Prism (version 10.5.0 (673), GraphPad) to obtain the IC_50_ values and confidence intervals at 95%. Experiments were performed independently twice, with a total of at least four replicates. Experiments with methyl viologen were performed at pH 6.8, while pH 7.5 was used for the other growth inhibition assays.

### Ethidium Efflux Assays

Ethidium efflux assays were performed like those previously described.^69^ In brief, *E. coli* ^Δ*acrB*Δ*emrE*Δ*mdfA*^ expressing wild-type EmrE, EmrE^W63^, and EmrE^E14D^ were grown to mid log phase at 37 °C (OD_600_ ∼ 1.0) in TBG medium. Cells were spun down and washed with PBS pH 7.0. After a second centrifugation step, the cell pellet was resuspended at OD_600_ = 0.6 and incubated with 10 μM CCCP and 25 μM ethidium bromide for 30 min at 37 °C while shaking. Cells were spun down, and the pellet was kept on ice, protected from light, until the fluorescence measurements. To measure efflux, cells were resuspended in PBS with 25 μM ethidium bromide. 0.2% glucose was added immediately before the fluorescence detection. The fluorescence decay was measured every 6 s for a total of 1800 s at 37 °C using a Molecular Devices FlexStation 3 instrument by exciting at 530 nm and detecting emission at 600 nm.

## Supplementary Material



## Notes

Proflavine and methyl viologen resistance assays, ethidium efflux assay, ^19^F NMR spectra for assignment of 6FW-labeled EmrE, MD simulation analyses, and ^19^F NMR spectra in the presence of ethidium bromide (Figures S1–S6) and theoretical vs experimental populations in heterodimers and the MD simulation setup (Tables S1 and S2) (PDF)

## +

Department of Biochemistry and Molecular Biology, Mayo Clinic, Rochester, MN 55905, USA
