# Supplementary material for: Quantification of Membrane Protein Conformational Free Energy from Mutations and a Single Atom
Source: J Am Chem Soc. 2025 Sep 15;147(38):34316–26. doi: 10.1021/jacs.5c04065 (PMC12464994; doi:10.1021/jacs.5c04065)
Supplement: Supplementary file 1 [file ja5c04065_si_001.pdf]

Supporting Information

Quantification of Membrane Protein Conformational Free Energy from Mutations and a Single Atom

Belen Ramirez-Cordero[1+] and Nathaniel J. Traaseth[1+*]

[1] Department of Chemistry, New York University, New York, NY 10003, USA

[+] Current address: Department of Biochemistry and Molecular Biology, Mayo Clinic, Rochester, MN 55905, USA

Contents

Figures S1-S6

Supplementary Tables S1-S2

References

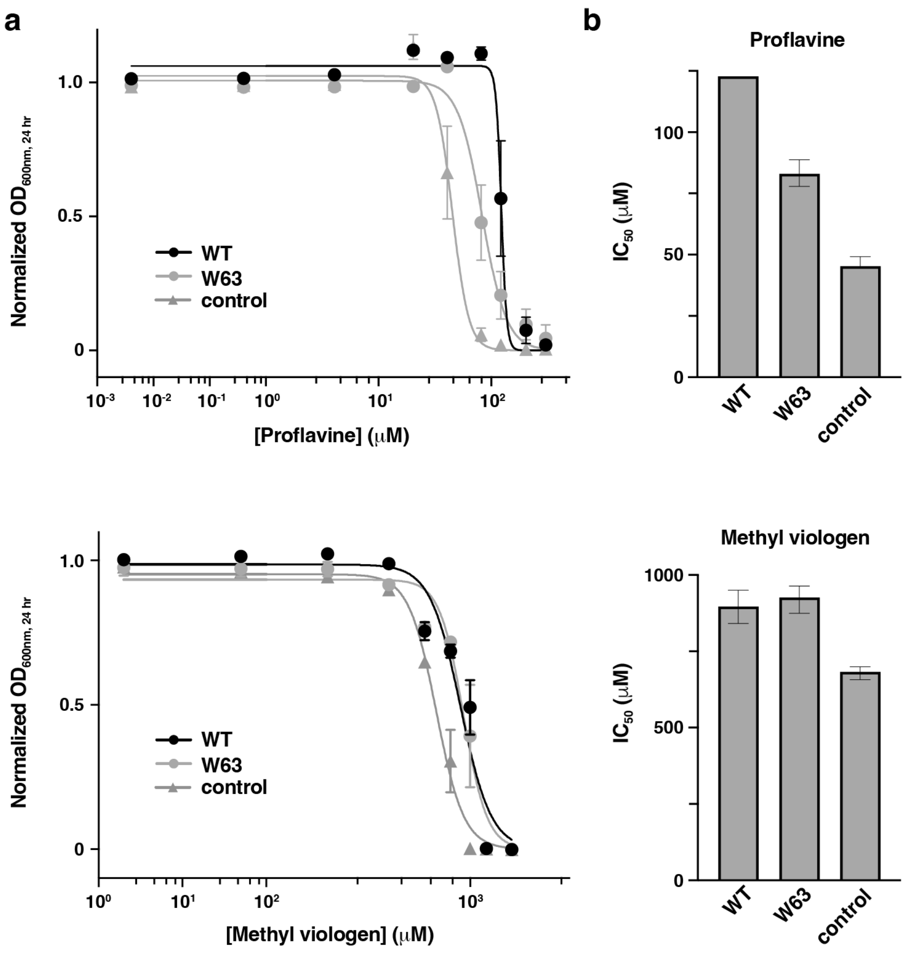

Figure S1. Growth inhibition assays against proflavine and methyl viologen.

a. Growth inhibition experiments against proflavine (top) and methyl viologen (bottom) in liquid culture of *E. coli*$^{\Delta acrB \Delta emrE \Delta mdfA}$ [1] expressing wild-type EmrE (WT), $EmrE^{W63}$ (W63), and a plasmid lacking the ribosome binding site (control). Error bars represent the standard deviation of measurements performed in triplicate. Relative growth was determined by comparing OD_{600nm} at 24 hr of growth at 37 °C against the corresponding growth without the compound tested. **b**. Inhibition concentration at 50% (IC_{50}) obtained from fitting the curves in the left panel, encompassing at least two independent experiments. Error bars represent the 95% confidence interval from data fitting. Proflavine IC_{50} values for WT (122 μM) and $EmrE^{W63}$ (83 μM) are statistically different from the control (45 μM, P-value < 0.0001). Methyl viologen IC_{50} values for

WT (898 μM) and $EmrE^{W63}$ (927 μM) are statistically different from the control (683 μM, P-value < 0.0001).

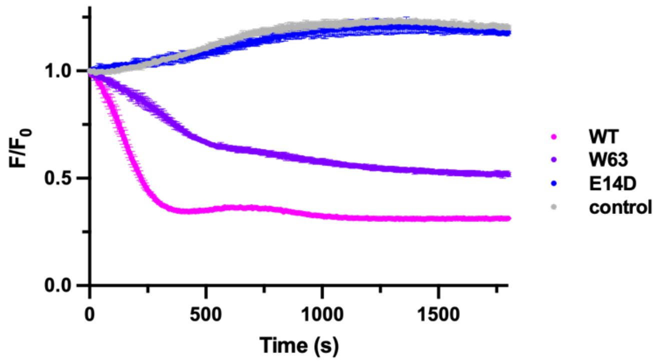

Figure S2. Ethidium efflux assays of EmrE^{W63}.

E. coli$^{\Delta acrB \Delta emrE \Delta mdfA}$ expressing wild-type EmrE, EmrE^{W63}, EmrE^{E14D}, and with a plasmid lacking the ribosome binding site (control) were subjected to an ethidium efflux assay. Bacteria accumulated with ethidium were initiated for efflux following addition of 0.2% glucose at Time = 0 s. All fluorescence data points were normalized to the starting fluorescence value. Error bars reflect the difference between duplicated experiments performed on the same day. The experiments were repeated at least twice with similar results.

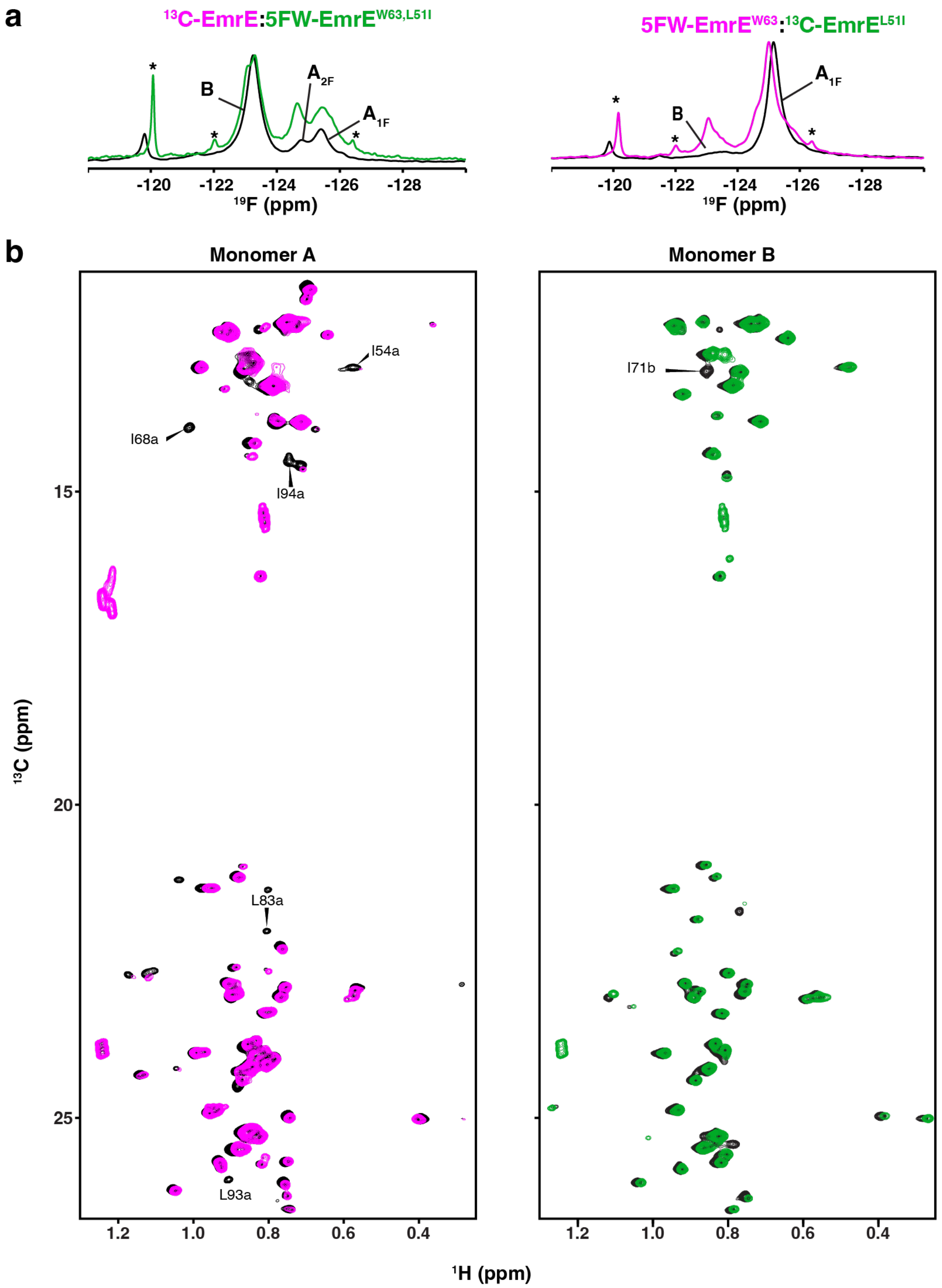

Figure S3. ^{19}F and ^{1}H/^{13}C HMQC spectra of heterodimers labeled with 5FW or ^{13}C in one monomer of the EmrE dimer.

^{19}F one-dimensional (**a**) and ^{1}H/^{13}C HMQC two-dimensional spectra (**b**) of heterodimers formed by mixing EmrE with the L51I mutant, where one monomer is 5FW-labeled (monomer mixed in

two-fold excess) and the other one is ^{13}C methyl-labeled at isoleucine, leucine, and valine residues. The double-labeled heterodimer spectra are displayed in green or magenta. As references for ^{19}F experiments, spectra of heterodimers $EmrE^{W63, L51I}$:5FW-$EmrE^{W63}$ (**a, left**) and 5FW-$EmrE^{W63, L51I}$:$EmrE^{W63}$ (**a, right**) are displayed in black. As references for $^{1}H/^{13}C$ HMQC experiments, spectra used to determine the EmrE proton-bound structure[2] for the heterodimer EmrE:$EmrE^{L51I}$ with monomer A (**b, left**) or monomer B (**b, right**) ^{13}C methyl-labeled are displayed in black. Since the double-labeled heterodimers were mixed with a two-fold excess of the 5FW-labeled protein, the ^{19}F spectra displayed more intense signals for the double fluorinated homodimers present due to statistical mixing compared to the reference spectra obtained with a three-fold excess of the unlabeled protein. The $^{1}H/^{13}C$ HMQC spectra of the double-labeled heterodimers display most of the signals in the reference spectra, albeit with a few missing peaks that are close to Trp63 in the structure.

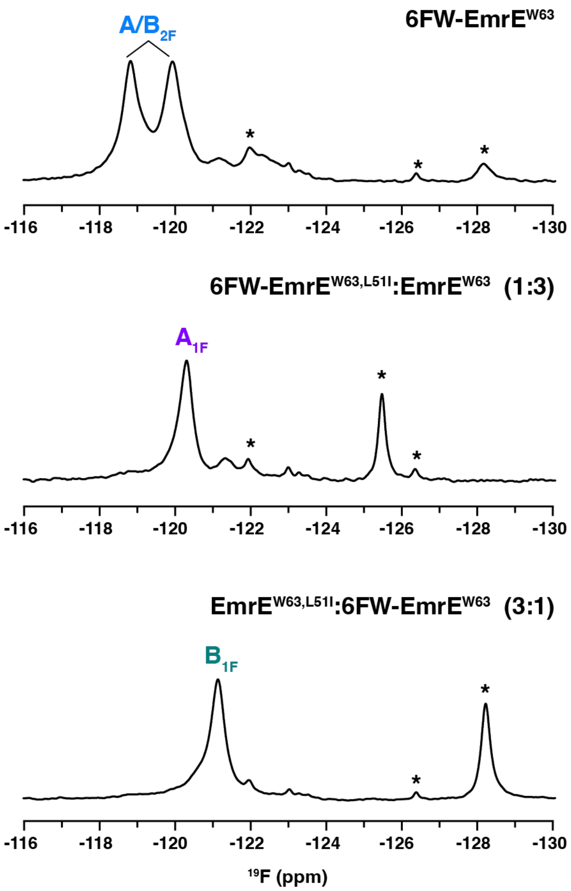

Figure S4. Monomer assignment of 6FW for residue Trp63 in EmrE.

One-dimensional ^{19}F NMR spectra of 6FW-EmrE^{W63} (top), 6FW-EmrE$^{W63,\ L51I}$:EmrE^{W63} (1:3) heterodimer (middle), and EmrE$^{W63,\ L51I}$:6FW-EmrE^{W63} (3:1) heterodimer (bottom) obtained at pH 3.5-4. These spectra were obtained at a lower pH to avoid overlap with the fluoride contaminant at -120 ppm at pH 5.6. Contaminants are indicated in the spectra with asterisks.

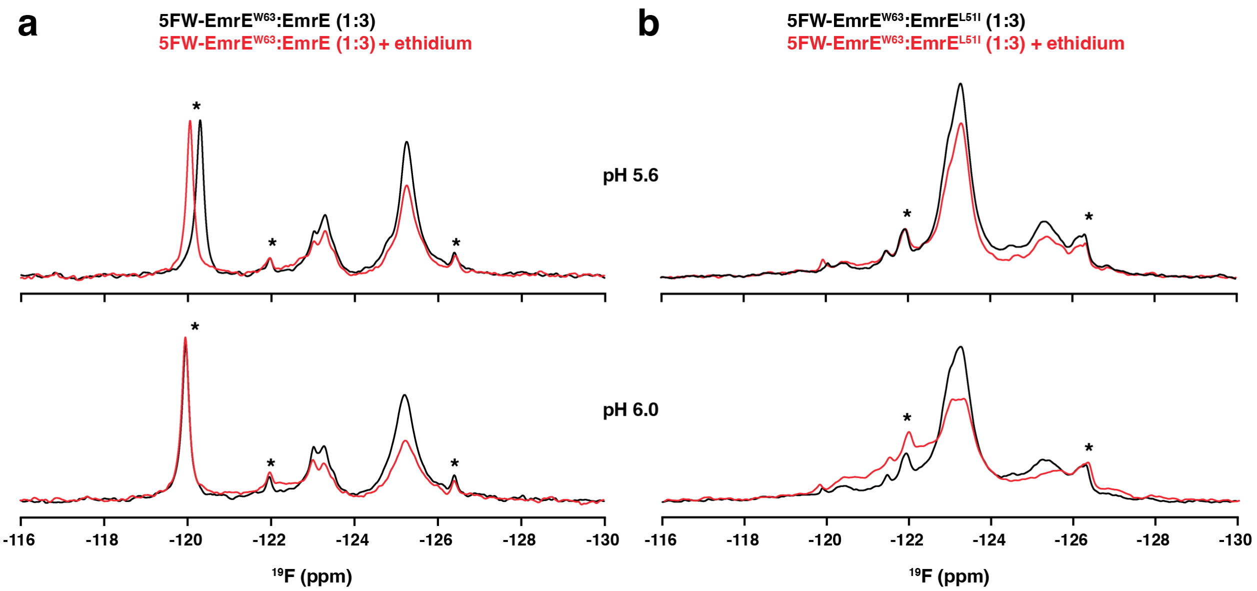

Figure S5. Effect of ethidium bromide addition to EmrE heterodimers.

Addition of 2-fold excess of ethidium bromide to heterodimer samples of 1:3 5FW-EmrE^{W63}:EmrE (a) or 1:3 5FW-EmrE^{W63}:EmrE^{L51I} (b). Heterodimers with and without ethidium are displayed in red and black, respectively. Spectra were acquired at two pH values indicated in the top row (pH 5.6) or bottom row (pH 6.0). Asterisks indicate fluorine contaminants derived from the lipids used to prepare the bicelles. In each panel, the addition of ethidium produced decreased peak intensities, suggesting faster conformational exchange and/or heterogeneity introduced upon substrate binding.

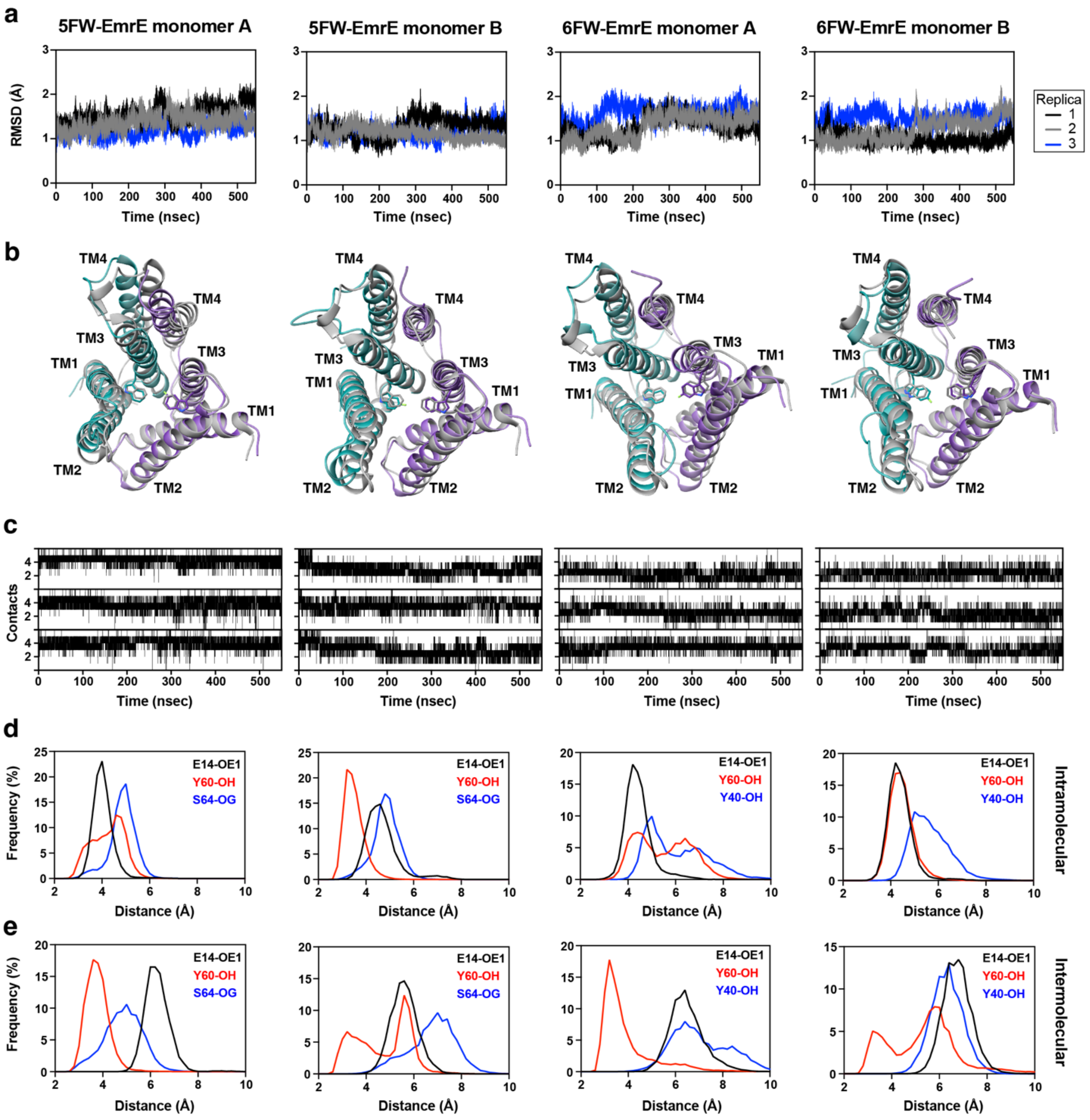

Figure S6. Analyses of MD simulations with 5FW or 6FW at Trp63 of EmrE.

a. RMSD analyses of MD simulations on the four simulations differing in the position of the fluorine label (i.e., 5FW or 6FW at Trp63 in monomer A or B). RMSD values of the backbone were calculated with respect to the starting structure (PDB ID: 8UWU). The three replicate simulations are colored as indicated on the right.

b. Representative snapshots of the MD simulations. The columns match with the labels displayed on top of panel a. These snapshots display the top view of EmrE structures with monomer A in purple and monomer B in teal superimposed with the NMR-derived structure of proton-bound EmrE (PDB:8UWU) in grey.

c. Number of contacts with hydrogen bond donors as a function of the simulation time. The columns match with the labels displayed on top of panel a. Results of three replicates are plotted in each panel.

d, e. Distance distribution between fluorine atom and the closest (d) intramolecular or (e) intermolecular oxygen atoms in the binding pocket of EmrE. Distance distributions were calculated from all replicate MD simulation runs. The columns match with the labels displayed on top of panel a.

Table S1. Calculated and experimental populations due to statistical mixing.

		Theoretical [a]				Experimental		
**Heterodimer**	**ratio**	$p_{A2F}=p_{B2F}$	p_{A1F}	p_{B1F}	**K**	$p_{A2F}=p_{B2F}$	p_{A1F}	p_{B1F}
5FW-EmrE^{W63}: EmrE^{W63}	1:1	0.2	0.473	0.127	0.268	0.186	0.468	0.159
5FW-EmrE^{W63}: EmrE^{W63}	1:3	0.071	0.676	0.181	0.268	0.060	0.659	0.220
5FW-EmrE$^{W63,\ L51I}$: EmrE^{W63}	1:3	0.067	0.782	0.018	0.023	ND	0.779	0.021
EmrE$^{W63,\ L51I}$: 5FW-EmrE^{W63}	3:1	0.067	0.185	0.615	3.33	0.072	0.181	0.620
5FW-EmrE$^{W63,\ E14Q}$: EmrE^{W63}	1:3	0.071	0.273	0.584	2.14	0.137	0.230	0.496
EmrE$^{W63,\ E14Q}$: 5FW-EmrE^{W63}	3:1	0.071	0.789	0.068	0.086	0.122	0.696	0.060

[a] Populations were determined considering that the sum of all the species is 1.

Table S2. MD simulation setup details

	**5FW-EmrE$_A$**	**5FW-EmrE$_B$**	**6FW-EmrE$_A$**	**6FW-EmrE$_B$**
Simulation box dimensions (Å)	90 x 90 x 120	90 x 90 x 120	90 x 90 x 120	90 x 90 x 120
Total atoms	53,986	53,404	53,440	53,560
Total waters	10,551	10,357	10,369	10,409
Salt concentration	20 mM NaCl	20 mM NaCl	20 mM NaCl	20 mM NaCl
Total lipids	160	160	160	160

References

(1) Tal, N.; Schuldiner, S. A coordinated network of transporters with overlapping specificities provides a robust survival strategy. *Proc Natl Acad Sci U S A* **2009**, *106* (22), 9051-9056. DOI: 10.1073/pnas.0902400106.

(2) Li, J.; Her, A. S.; Besch, A.; Ramirez-Cordero, B.; Crames, M.; Banigan, J. R.; Mueller, C.; Marsiglia, W. M.; Zhang, Y.; Traaseth, N. J. Dynamics underlie the drug recognition mechanism by the efflux transporter EmrE. *Nat Commun* **2024**, *15* (1), 4537. DOI: 10.1038/s41467-024-48803-2 From NLM Medline.
